# Protective Role of Proton-Sensing TDAG8 in Lipopolysaccharide-Induced Acute Lung Injury

**DOI:** 10.3390/ijms161226145

**Published:** 2015-12-04

**Authors:** Hiroaki Tsurumaki, Chihiro Mogi, Haruka Aoki-Saito, Masayuki Tobo, Yosuke Kamide, Masakiyo Yatomi, Koichi Sato, Kunio Dobashi, Tamotsu Ishizuka, Takeshi Hisada, Masanobu Yamada, Fumikazu Okajima

**Affiliations:** 1 Laboratory of Signal Transduction, Institute for Molecular and Cellular Regulation, Gunma University, Maebashi 371-8512, Japan; cmogi@gunma-u.ac.jp (C.M.); a-haruka@gunma-u.ac.jp (H.A.-S.); mtobo@gunma-u.ac.jp (M.T.); kosato@gunma-u.ac.jp (K.S.); 2 Department of Medicine and Molecular Science, Gunma University Graduate School of Medicine, Maebashi 371-8511, Japan; m08702012@gunma-u.ac.jp (Y.K.); m09702007@gunma-u.ac.jp (M.Y.); myamada@gunma-u.ac.jp (M.Y.); 3 Gunma University Graduate School of Health Sciences, Maebashi 371-8514, Japan; dobashik@gunma-u.ac.jp; 4 Third Department of Internal Medicine, Faculty of Medical Sciences, University of Fukui, Fukui 910-1193, Japan; tamotsui@u-fukui.ac.jp

**Keywords:** acute lung injury, TDAG8, lipopolysaccharide, neutrophil, KC

## Abstract

Acute lung injury is characterized by the infiltration of neutrophils into lungs and the subsequent impairment of lung function. Here we explored the role of *TDAG8* in lung injury induced by lipopolysaccharide (LPS) administrated intratracheally. In this model, cytokines and chemokines released from resident macrophages are shown to cause neutrophilic inflammation in the lungs. We found that LPS treatment increased *TDAG8* expression in the lungs and confirmed its expression in resident macrophages in bronchoalveolar lavage (BAL) fluids. LPS administration remarkably increased neutrophil accumulation without appreciable change in the resident macrophages, which was associated with increased penetration of blood proteins into BAL fluids, interstitial accumulation of inflammatory cells, and damage of the alveolar architecture. The LPS-induced neutrophil accumulation and the associated lung damage were enhanced in *TDAG8*-deficient mice as compared with those in wild-type mice. LPS also increased several mRNA and protein expressions of inflammatory cytokines and chemokines in the lungs or BAL fluids. Among these inflammatory mediators, mRNA and protein expression of KC (also known as CXCL1), a chemokine of neutrophils, were significantly enhanced by *TDAG8* deficiency. We conclude that TDAG8 is a negative regulator for lung neutrophilic inflammation and injury, in part, through the inhibition of chemokine production.

## 1. Introduction

Acute lung injury (ALI) and its severe form, acute respiratory distress syndrome (ARDS), are caused by several disorders, including pneumonia, sepsis, and aspiration of gastric contents, and are characterized by pulmonary edema due to increased permeability of the alveolar epithelial and endothelial barriers and the subsequent impairment of arterial oxygenation. Despite a variety of efforts to treat the disorders, morbidity and mortality remain high [1,2,3]. The accumulation of neutrophils in the lung microvasculature, interstitium, and bronchoalveolar space is believed to play a key role in ALI and ARDS [1,2,3]. Neutrophils secrete potent antibacterial molecules, including proteases, cationic compounds, and reactive oxidants; however, dysregulation of this innate inflammatory response leads to damage of the alveolar barrier function, thereby inducing respiratory failure [1,3].

Extracellular pH is strictly maintained at 7.35 to 7.45 in the blood. The microvasculature system is well developed in the lungs, and the pH in the interstitium is thought to be maintained at the same level as in the blood. On the other hand, in lung lining fluids, such as airway surface fluid and alveolar subphase fluid, the pH appears to be around 6.9 in order to defend the host against invading bacteria [4]. Although the precise mechanism of the acid–base balance in the airway and alveolar subphase fluids is not fully understood, alveolar epithelial cells and macrophages may contribute to the mildly acidic pH of lung lining fluids [4]. The pH of the interstitium and the bronchoalveolar space has been shown to fall in inflammatory diseases, such as ARDS. Indeed, the pH of the exhaled breath condensate decreased to less than 6.0 in ventilated patients with ARDS and ALI as compared to volunteers [5].

Recent studies have shown that proton-sensing ovarian cancer G protein-coupled receptor 1 (*OGR1*) family G protein-coupled receptors (GPCRs), including *OGR1* (also known as *GPR68*), G protein-coupled receptor 4 (*GPR4*), and T-cell death-associated gene 8 (*TDAG8* or *GPR65*), mediate cellular actions induced by alkaline and acidic pH of 8 to 6 through histidine residues in a variety of cell types [6,7,8]. *TDAG8* is mainly expressed in bone marrow-derived or hematopoietic cells [9] and has been shown to mediate the inhibition of extracellular acidification-induced inflammatory cytokine production in macrophages [10], the inhibition of superoxide anion production in neutrophils [11], and the survival response in eosinophils [12]. In the present study, we explored the role of proton-sensing *TDAG8* in lung injury as induced by intratracheally administrated lipopolysaccharide (LPS) and found that TDAG8 is protective against lung neutrophilic inflammation and injury.

## 2. Results

### 2.1. Expression Profiles of Proton-Sensing GPCRs in Lung Tissues and Bronchoalveolar Lavage (BAL) Fluid Cells

**Figure 1:**
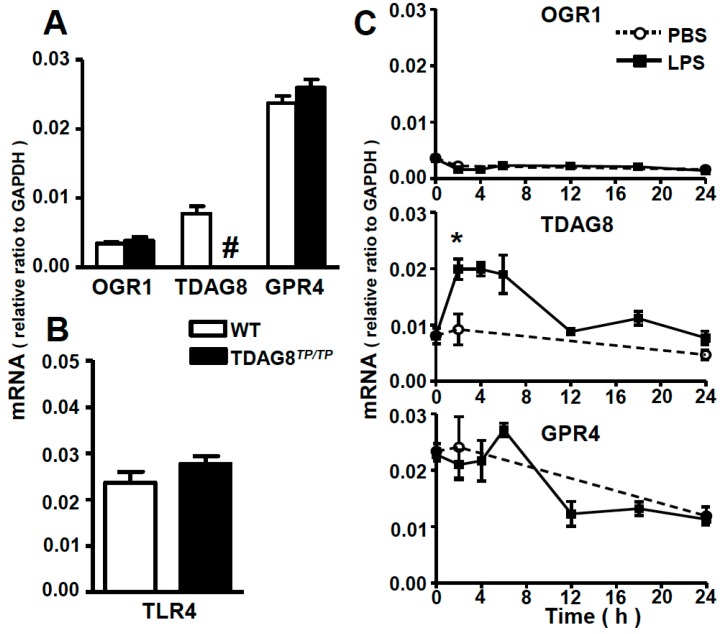
*TDAG8* mRNA expression is enhanced by LPS treatment. The mRNA expressions of proton-sensing GPCRs in (**A**) and TLR4 in (**B**) in lung tissues of WT and *TDAG8^TP/TP^* mice were measured. ^#^ The expression of *TDAG8* is undetectable; (**C**) The time course change in mRNA expression of proton-sensing GPCRs in lung tissues after PBS or LPS instillation. Results are expressed as a ratio relative to GAPDH. Data are mean ± SEM of *n* = 3–5 for each group. The effect of LPS is significant, * *p* < 0.05 (PBS *vs.* LPS).

To determine the expression profile of proton-sensing GPCRs, we measured the mRNA expression because of the lack of their specific antibodies. Consistent with previous results [13], the mRNAs of proton-sensing GPCRs, including *OGR1*, *TDAG8*, and *GPR4*, are expressed in lung tissues (Figure 1A). *GPR4* is the highest proton-sensing GPCR. We also confirmed that *TDAG8^TP/TP^* mice show a complete loss of *TDAG8* expression but no change in other proton-sensing GPCR expression (Figure 1A). Moreover, mRNA and protein expressions of TLR4, a receptor for LPS, (Figure 1B and Figure S1) and mRNA of CD14, a co-receptor of LPS, (Figure S2) were not appreciably affected by *TDAG8* deficiency. The effects of intratracheal administration of LPS on the expression of proton-sensing GPCRs in lung tissues are shown in Figure 1C. While *OGR1* and *GPR4* mRNA expression were hardly affected by LPS treatment, *TDAG8* mRNA expression nearly doubled 2 h after LPS treatment (Figure 1C).

**Figure 2:**
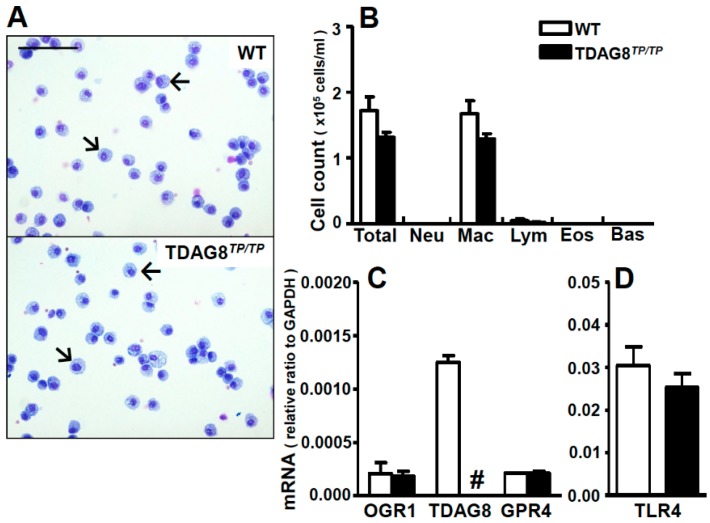
Macrophages are major cell types expressing *TDAG8* in BAL fluids. (**A**) Representative cell images of BAL fluids of WT and *TDAG8^TP/TP^* mouse. Arrows represent macrophages. Scale bar is 100 µm; (**B**) Differential cell counts in BAL fluids. Total, total white blood cells; Neu, neutrophils; Mac, macrophages; Lym, lymphocytes; Eos, eosinophils; and Bas, basophils. Data are mean ± SEM of *n* = 6 for each group; (**C**,**D**) The mRNA expression profile of proton-sensing GPCRs and TLR4 in BAL fluid cells. ^#^ The expression of *TDAG8* was undetectable. Eight to 10 mice were used in each set and data are mean ± SEM of three separate experiments.

We next performed BAL to identify major cell types and the expression profiles of proton-sensing GPCRs in the bronchoalveolar space. Differential cell counts showed that cell types present in BAL fluids are macrophages, and other cell types, including neutrophils, lymphocytes, eosinophils, and basophils, are not detectable or are less than 1% of the total cells in either WT mice or *TDAG8^TP/TP^* mice (Figure 2A,B). The expression profile of proton-sensing GPCRs in BAL fluid cells is shown in Figure 2C. In contrast to the mRNA profile in the lung, where the *GPR4* expression level was the highest among proton-sensing GPCRs (Figure 1A), *TDAG8* was a major proton-sensing GPCR in the cells of BAL fluids (Figure 2C). Together, these results suggest that *TDAG8*-expressing cells in the bronchoalveolar space are macrophages and, moreover, that *GPR4*-expressing cells in the lung are mainly structural cells, such as alveolar epithelial cells and endothelial cells. Supporting this contention is the fact that, regardless of species differences, airway smooth muscle cells, airway epithelial cells, and vascular endothelial cells express *OGR1* and/or *GPR4* but not *TDAG8* (Figure S3). We also confirmed that macrophages in BAL fluids from *TDAG8^TP/TP^* mice are lacking in *TDAG8* expression (Figure 2C), without changes in the mRNA expression of other proton-sensing GPCRs and TLR4 (Figure 2D) and the protein expression of TLR4 (Figure S1). Moreover, the mRNA expression of CD14 was not appreciably affected by *TDAG8* deficiency (Figure S2). We then focused on the role of *TDAG8* in LPS-induced ALI.

### 2.2. Enhancement of LPS-Induced Neutrophil Accumulation and Lung Injury by TDAG8 Deficiency

**Figure 3:**
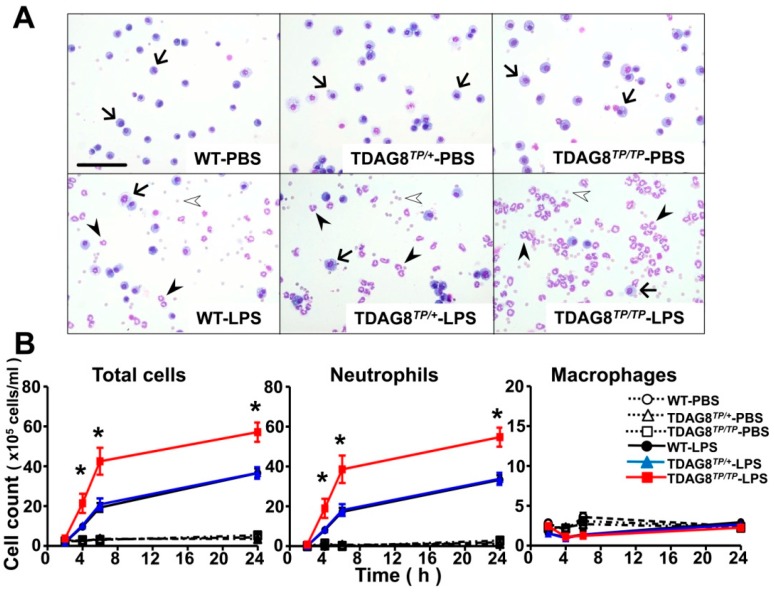
*TDAG8* deficiency enhanced LPS-induced neutrophil accumulation. (**A**) Representative images of cells in BAL fluids 6 h after PBS or LPS treatment in WT, *TDAG8^TP/+^* and *TDAG8^TP/TP^* mice. Arrows, arrowheads, and open arrowheads represent macrophages, neutrophils, and erythrocytes, respectively. Scale bar is 100 µm. One tenth volume of BAL fluids was used for LPS treatment as compared with those for PBS treatment; (**B**) Time course change in differential cell counts in BAL fluids. It is noted that, in addition to neutrophils, erythrocytes are also detected reflecting the damage of lung vasculature in BAL fluids from LPS-treated mice. However, erythrocytes were neglected for differential cell counts. Total cells, total white blood cells. Data are mean ± SEM of *n* = 5–15 for each group. The effect of *TDAG8* deficiency is significant, * *p* < 0.05 (*TDAG8^TP^*^/*TP*^-LPS *vs.* WT-LPS or *TDAG8^TP^*^/+^-LPS).

Neutrophil accumulation is a cardinal feature of ALI. We analyzed the numbers and population of cells present in BAL fluids in LPS-treated mice (Figure 3). In the absence of LPS treatment, as shown in Figure 2A as well, the major cell types are macrophages, regardless of *TDAG8* expression levels, and neutrophils are remarkably accumulated with LPS treatment (Figure 3A). The time course of the accumulation of cells in BAL fluids is shown in Figure 3B. The total number of white blood cells is remarkably increased from 4 h after the treatment with a 2-h lag time, becoming maximal at 24 h. Differential cell counts revealed the prominent recruitment of neutrophils, and the neutrophil accumulation into BAL fluids was significantly enhanced in *TDAG8*-deficient mice as compared with WT and heterozygous mutant mice (Figure 3B). However, an appreciable change in the number of resident macrophages was not observed, and the number of white blood cells other than macrophages and neutrophils was very small, even after treatment with LPS.

**Figure 4:**
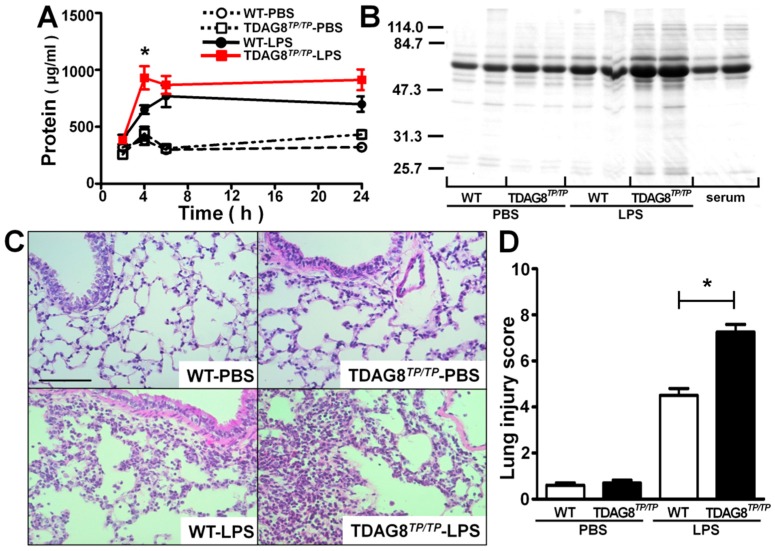
The LPS-induced protein penetration and lung injury are greater in *TDAG8*-deficeient mice than in WT mice. (**A**) BAL fluid samples were collected at the indicated time after PBS or LPS instillation for measurement of protein concentration. Data are mean ± SEM of *n* = 12–17 for each group. The effect of *TDAG8* deficiency is significant, * *p* < 0.05 (*TDAG8^TP/TP^*-LPS *vs.* WT-LPS); (**B**) Proteins of BAL fluid sample (duplicate for each group) were separated by SDS-PAGE and stained with Coomassie brilliant blue. Mouse serum at two different concentrations (right two lanes) was used as standard; (**C**) Histological analysis of lung sections was performed 4 h after PBS or LPS instillation. Three representative lung sections per mouse, from 5–10 mice, are shown for each group. Scale bar is 100 µm; (**D**) Lung injury scores by analysis of histopathological sections from lungs 4 h after PBS or LPS instillation. Data are mean ± SEM of *n* = 5–10 for each group. The effect of *TDAG8* deficiency is significant, * *p* < 0.05 (*TDAG8^TP^*^/*TP*^-LPS *vs.* WT-LPS).

The neutrophil accumulation was accompanied by protein penetration into BAL fluids (Figure 4A). Similarly to neutrophil infiltration, significant protein penetration by LPS was observed at 4 h with a 2-h lag time (Figure 4A). The increased proteins may be derived from blood, as evidenced by the finding that albumin is a major protein in BAL fluids (Figure 4B). The LPS-induced protein penetration is enhanced by *TDAG8* deficiency (Figure 4A,B), suggesting that the barrier function of the vascular system was more severely injured by LPS treatment in *TDAG8*-deficient mice than in WT mice. Consistent with the protein penetration results, LPS treatment caused interstitial accumulation of inflammatory cells, probably neutrophils, and injured the bronchial architecture in WT mice (Figure 4C,D). LPS-induced lung injury was greater in *TDAG8*-deficient mice than in WT mice, whereas there was no appreciable change in the bronchial architecture by *TDAG8* deficiency in the basal state without LPS treatment. These results suggest that TDAG8 is protective against LPS-induced ALI.

### 2.3. Involvement of TDAG8 in the Regulation of Cytokine and Chemokine Expression in Lungs

**Figure 5:**
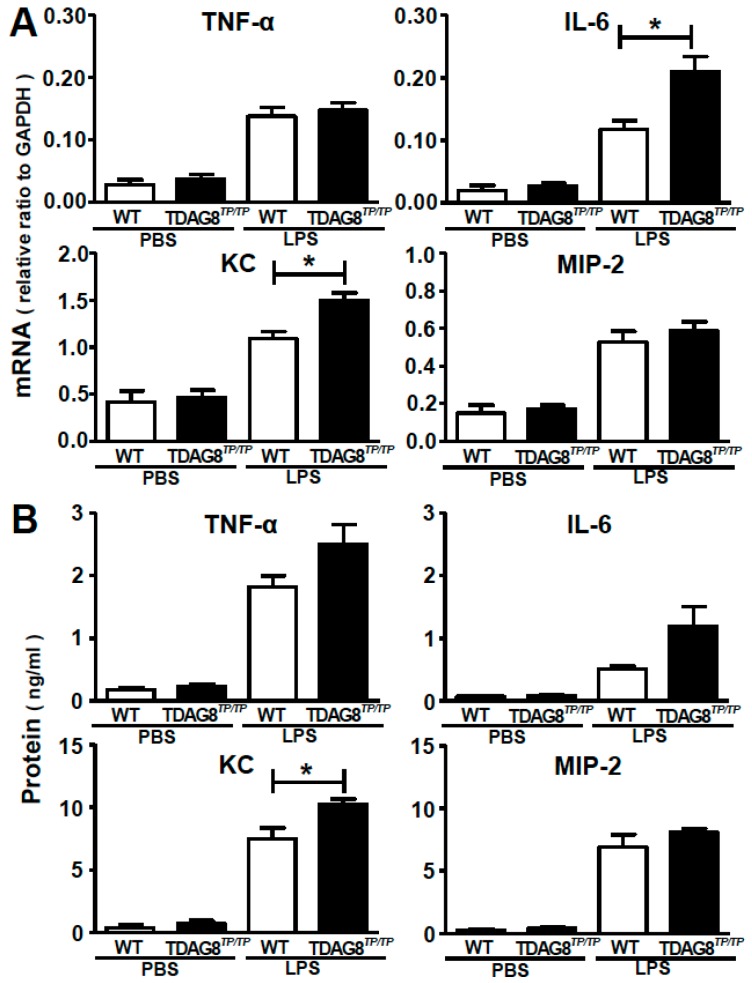
*TDAG8* is involved in the regulation of LPS-induced cytokine and chemokine expression. Mice (WT and *TDAG8^TP/TP^*) were treated with PBS or LPS as described in Figure 3. Cytokine (TNF-α, IL-6) and chemokine (KC/CXCL1, MIP-2/CXCL2) expression levels were evaluated 2 h after intratracheal instillation. (**A**) The mRNA expression of the lungs. Results are expressed as relative ratio to GAPDH; (**B**) The cytokine and chemokine protein level of the BAL fluids. Results are expressed as ng proteins/mL. Data are mean ± SEM of *n* = 4–10 for each group. The effect of *TDAG8* deficiency is significant, * *p* < 0.05 (*TDAG8^TP/TP^*-LPS *vs.* WT-LPS).

It is well known that neutrophil infiltration and lung inflammation in ALI are associated with cytokine and chemokine expression [3]. As shown in Figure 5, LPS treatment increased the mRNA expression of cytokines, including TNF-α and IL-6, and chemokines, including KC (also known as CXCL1) and MIP-2 (also known as CXCL2), both of which are known to recruit neutrophils, in the lung even 2 h after treatment (Figure 5A). The early expression of IL-6 and KC mRNA was significantly enhanced by *TDAG8* deficiency. Consistent with mRNA expression, the protein levels of KC in BAL fluids were significantly enhanced 2 h after LPS treatment by *TDAG8* deficiency (Figure 5B). As for IL-6, although the effect of *TDAG8* deficiency is not significant, it tends to increase IL-6 protein expression in BAL fluids (Figure 5B). On the other hand, the effects of *TDAG8* deficiency on the protein levels of TNF-α and MIP-2 were insignificant in the same sample.

## 3. Discussion

In the present study, we have for the first time shown that *TDAG8* is protective against intratracheally administrated LPS-induced ALI. This LPS model is a well-characterized animal model of ALI that occurs by Gram-negative bacterial infection [14]. Both alveolar macrophages and epithelial cells are the first cells to encounter LPS when the endotoxin is administrated by intra-alveolar routes [15]. The endotoxin would then penetrate into the interstitium and the microvasculature. In these cell types, TLR4 is expressed, and LPS stimulates the expression of proinflammatory cytokines, including TNF-α, IL-6, and IL-1β, and chemokines, including KC [16,17,18]. However, it is reported that alveolar macrophages but not structural cells, such as endothelial cells and epithelial cells in the lungs, are responsible for the recruitment of neutrophils to the lungs and the expression of inflammatory genes when LPS is administrated by intra-alveolar routes, as in the present study [19], whereas endothelial cells rather than leukocytes seem to be important for the neutrophil infiltration in the case of systemic LPS administration [20]. Thus, resident macrophages may be responsible for LPS-induced lung injury in our experimental model.

Our results showed that *TDAG8* deficiency enhances LPS-induced lung injury in association with increased KC and IL-6 mRNA expression and KC release as early as 2 h after LPS stimulation. Since KC is a potent chemoattractant of neutrophils, increased neutrophil accumulation may be partly explained by the increased level of KC in *TDAG8*-deficient mice. Serum IL-6 is increased in patients with acute kidney injury and ALI, and it has been thought to be a biomarker of a poor outcome of lung injuries [21]. A recent study has shown that increased serum IL-6 in acute kidney injury animal model caused lung injury by increasing lung endothelial KC production and subsequent neutrophil infiltration [22], suggesting that IL-6 is a pathogenic mediator of lung injury that plays a role in neutrophil infiltration through KC production. Thus, enhanced KC and IL-6 expression in *TDAG8*-deficient mice may be closely related to neutrophil infiltration. Which cells are primarily important for the TDAG8-dependent enhancement of KC and/or IL-6 expression? *TDAG8* expression is limited in bone marrow-derived or hematopoietic cells, such as macrophages [9]. Indeed, macrophages and neutrophils express *TDAG8* (Figure S3). Although neutrophils express *TDAG8* and have the ability to express these cytokines and chemokines in response to LPS [23], neutrophil accumulation was not observed until 4 h after LPS treatment (Figure 3B). These results suggest that the enhancement of chemokine and cytokine expression by *TDAG8* deficiency in the lungs at the early time point (2 h) after LPS administration reflects the inhibitory action of *TDAG8* on TLR4-mediated gene regulation in macrophages but not in neutrophils. We have previously shown that macrophages respond to acidic pH, resulting in the inhibition of LPS-induced inflammatory cytokines, including IL-6, in a manner dependent on *TDAG8* expression [10]. Unfortunately, however, we have not yet proven the *TDAG8* dependency on LPS-induced KC production in isolated peritoneal and alveolar macrophages. Further studies are needed to identify the cell types involved in the enhancement of KC and IL-6 expression by *TDAG8* deficiency.

Although our results suggest that neutrophils may not be involved in TDAG8 regulation of KC expression at the early time point after LPS treatment, this does not necessarily rule out a possible role of the TDAG8 in neutrophils in their accumulation in the lungs. That may involve a variety of events, including migration, adhesion to endothelial cells, infiltration into the interstitium, and survival. The migration response to chemokines is only a part of these events. Moreover, neutrophils secrete proteases, a variety of cationic polypeptides, and reactive oxygen species, such as the superoxide anion, resulting in lung damage [1]. The production of superoxide anion in neutrophils has been shown to be suppressed by acidic pH through TDAG8 [11]. This anti-inflammatory role of TDAG8 in neutrophils might also be involved in the exacerbation of lung injury in *TDAG8*-deficient mice. In any event, in a future study, we should focus on the role of TDAG8 in the regulation of neutrophil functions.

In lung injury and inflammation, OGR1 seems to be involved in the dendritic cell migration to lymph nodes, thereby inducing Th2 cytokine production and cardinal features of asthmatic responses, including eosinophilic inflammation and airway hyperresponsiveness in the ovalbumin-induced asthma model [13]. OGR1 mediates mildly acidic pH-induced IL-6 and connective tissue growth factor (CTGF) expression [24,25], Ca^2+^ mobilization [24], and constriction [26] in airway smooth muscle cells. OGR1 in bronchial epithelial cells has been shown to mediate mucin production in response to acidic pH [27]. As for GPR4, although its role in respiratory systems has not been examined, this GPCR is expressed in vascular endothelial cells [28] and may be involved in the monocyte adhesion and penetration in inflammation [28,29]. Thus, *OGR1* and *GPR4* are expressed in a variety of cell types assumed to participate in lung physiology and pathophysiology and seem to function as inflammatory proton-sensing receptors. On the other hand, *TDAG8*, the expression of which increased in response to Gram-negative bacterial surface LPS (Figure 1C), seems to function as a negative regulator of inflammation in lung defense systems. Supporting the anti-inflammatory role of TDAG8, this receptor knockout has been reported to exacerbate inflammation in the anti-type II collagen antibody-induced arthritis model, possibly through the changing functions of neutrophils and macrophages [30], and to stimulate bone resorption in ovariectomized mice though a change in osteoclast function [31], although the survival role of TDAG8 in eosinophils augments rather than attenuates lung inflammation in asthma models [12]. Further studies are necessary to clarify the roles of proton-sensing GPCRs in lung function, which may provide novel therapeutic targets for respiratory disorders, including ALI and asthma.

## 4. Experimental Section

### 4.1. Animals

All animal procedures were performed in accordance with the guidelines of the Animal Care and Experimentation Committee of Gunma University (Permit Number: 14-029, 18 June 2014). *TDAG8^TP^*^/*TP*^ mouse constructed by the *Sleeping Beauty* transposon system [32] was provided by Drs. K. Horie and J. Takeda of Osaka University, Dr. Takao Shimizu of Tokyo University, and Dr. Satoshi Ishii of Akita University, and was backcrossed for eight generations with C57BL/6 mice [10]. Female C57BL/6 *TDAG8^+/+^* (WT), heterozygous (*TDAG8^TP^*^/*+*^), or gene-deficient (*TDAG8^TP^*^/*TP*^) mice were generated by heterozygous brother–sister mating and used at 8–10 weeks of age. Mice were under specific pathogen-free conditions. The *TDAG8* genes were identified by PCR genotyping using the previously described primers [10].

### 4.2. Murine Model of ALI

ALI was induced by intratracheal administration of lipopolysaccharides (LPS) from *Escherichia coli* 0111:B4 (L4391, Sigma-Aldrich, St. Louis, MO, USA) at a dose of 2.5 mg/kg [33,34]. In brief, mice were anesthetized, which was followed by intratracheal instillation of sterile phosphate-buffered saline (PBS) or LPS in 50 μL PBS with a 23-gauge catheter using the tongue-pull maneuver. At 2, 4, 6, and/or 24 h after administration, biological and histological analyses were performed as follows.

### 4.3. Bronchoalveolar Lavage (BAL)

Mice were given a lethal dose of sodium pentobarbital (60 mg/kg i.p.). BAL was performed by cannulating the trachea with a 23-gauge catheter and lavaging bronchi and alveoli five times with 600 μL of sterile PBS solution. The lavage fluids were centrifuged (300× *g* for 15 min), and the supernatant of the BAL fluid was stored at −80 °C until the subsequent assay. The cell pellet was resuspended in 1 mL of PBS and total cells were counted in a Neubauer chamber. Differential cell counts were performed by cytospin preparations and subsequent staining with May–Giemsa stain (May–Grünwald’s eosin–methylene blue solution and Giemsa’s azur eosin-methylene blue solution, Merck KGaA, Darmstadt, Germany). Then, neutrophils, macrophages, lymphocytes, eosinophils, and basophils were counted as previously described [13,35]. The protein concentration in BAL fluid was measured by Pierce BCA protein assay kit (Thermo Fisher Scientific, Asheville, NC, USA) according to the manufacturer’s recommendations, as previously described [36].

### 4.4. Electrophoresis of BAL Fluids

Proteins in BAL fluids were separated by sodium dodecyl sulfate polyacrylamide gel electrophoresis (SDS-PAGE). The samples were diluted with the sample buffer containing 50 mM Tris buffer (pH = 7.4), 1% SDS, 2% 2-mercaptoethanol, and 2.5% glycerol and processed to SDS-PAGE with 4.2% stacking and 10% resolving gels at constant current of 25 mA per gel. Following electrophoresis, the gels were stained with 0.25% Coomassie brilliant blue (CBB-R250, WAKO, Osaka, Japan).

### 4.5. Measurement of Cytokines and Chemokines

Cytokine and chemokine levels in BAL fluids were measured by commercially available DuoSet ELISA development kits (R&D Systems, Minneapolis, MN, USA) for KC/CXCL1, MIP-2/CXCL2, IL-6, and TNF-α, according to the manufacturer’s instructions.

### 4.6. Histological Studies

The left lungs were fixed with 10% formalin that was neutrally buffered overnight. The tissue fixed with formalin was washed with PBS and dehydrated in 70% ethanol before paraffin embedding. The sections (5 μm) were stained with hematoxylin and eosin (Sakura Finetek, Tokyo, Japan) and examined by light microscopy. The severity of the tissue was evaluated by independently scoring four parameters, *i.e.*, alveolar congestion, hemorrhage, aggregation of neutrophil or leukocyte infiltration, and thickness of the wall, in a blind manner based on the methods described previously [37,38]. Then each parameter was scored on a scale of 0–4. A score of 0, no lung abnormalities; 1, lesions involving less than 25% of the lung; 2, lesions involving 25%–50% of the lung; 3, lesions involving 50%–75% of the lung; and 4, lesions involving more than 75% of the lung. The total lung injury score was expressed as the summation of the all scores.

### 4.7. Measurement of mRNAs

Total RNA of the lungs and cells was isolated using RNAiso Plus (TaKaRa-bio, Siga, Japan) based on the instructions from the manufacturer. After treatment with DNaseI (Promega, Madison, WI, USA) to remove traces of genomic DNA possibly contaminating the RNA preparations, 2 μg of the purified RNA was reverse-transcribed according to the manufacturer’s recommendations (Applied Biosystems, Foster City, CA, USA). The expression of mRNAs was measured by quantitative real-time TaqMan PCR with Mx3000P (Agilent Technologies, Santa Clara, CA, USA) as described previously [10,39,40]. The primers for the mouse *OGR1* (Mm01335272), *TDAG8* (Mm00433695), *GPR4* (Mm00558777), *CXCL1* (or KC; Mm04207460), *CXCL2* (or MIF-2; Mm00436450), *TNF-α* (Mm00443258), *IL-6* (Mm00446190), *TLR4* (Mm00445274), and *GAPDH* (4352932E) were purchased from Applied Biosystems.

### 4.8. Statistical Analysis

The results are presented as the mean ± SEM. In *in vivo* experiments, we usually used three to five mice per group in each experiment and carried out the same experiments at least three times. All the results were combined for the presentation of the results of *in vivo* study, unless otherwise stated. One-way ANOVA and Tukey test for *post hoc* comparisons were used to determine differences between control and experimental groups. Parameter changes between different groups over time were evaluated by a two-way ANOVA and Bonferroni post-tests to compare replicate means. GraphPad Prism 5 (La Jolla, CA, USA) was used for the statistical calculation. A *p* value of less than 0.05 was considered significant.

## 5. Conclusions

TDAG8 plays a protective role against lung neutrophilic inflammation and injury, in part by inhibiting the expression of KC, a chemokine for neutrophils, and the subsequent infiltration of neutrophils into the lungs. *TDAG8* is expressed in resident macrophages, which may be at least partly responsible for the regulation of KC expression. *TDAG8* is also expressed in neutrophils; however, its role in the cell functions is currently unknown.

## Supplementary Materials

Supplementary materials can be found at http://www.mdpi.com/1422-0067/16/12/26145/s1.
