# Supplementary material for: Protective Role of Proton-Sensing TDAG8 in Lipopolysaccharide-Induced Acute Lung Injury
Source: Int J Mol Sci. 2015 Dec 4;16(12):28931–42. doi: 10.3390/ijms161226145 (PMC4691092; doi:10.3390/ijms161226145)
Supplement: Supplementary file 1 [file ijms-16-26145-s001.pdf]

Supplementary Materials: Protective Role of Proton-Sensing TDAG8 in Lipopolysaccharide-Induced Acute Lung Injury

Hiroaki Tsurumaki, Chihiro Mogi, Haruka Aoki-Saito, Masayuki Tobo, Yosuke Kamide, Masakiyo Yatomi, Koichi Sato, Kunio Dobashi, Tamotsu Ishizuka, Takeshi Hisada, Masanobu Yamada and Fumikazu Okajima

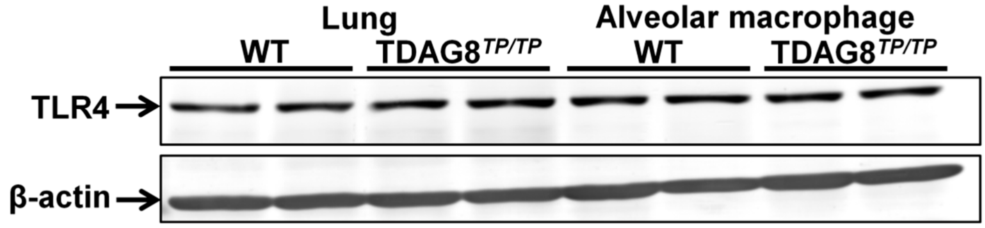

Figure S1. No appreciable effect of TDAG8 deficiency on TLR4 protein expression. The proteins were extracted from lung tissues and alveolar macrophages in WT and TDAG8-deficient mice with a lysis buffer composed of 20 mM Tris–HCl, pH = 8.0, 150 mM NaCl, 1mM EDTA–PBS, 0.1% sodium dodecyl sulfate, 0.5% sodium deoxycholate, 1% Nonidet P-40, and 1% proteinase inhibitor cocktail (Sigma-Aldrich, St. Louis, MO, USA). The lysate was centrifuged at 14,000× *g* for 20 min. The cell extracts were then subjected to Western blotting analysis using sodium dodecyl sulfate–polyacrylamide gel electrophoresis for detection of TLR4 and β-actin proteins. Protein bands were detected by alkaline phosphatase method. The primary antibodies were obtained from Sigma-Aldrich (St. Louis, MO, USA) for anti-β-actin, and from Santa Cruz Biotechnologies (Dallas, TX, USA) for anti-TLR4. Two different batches of the tissues and cells were used.

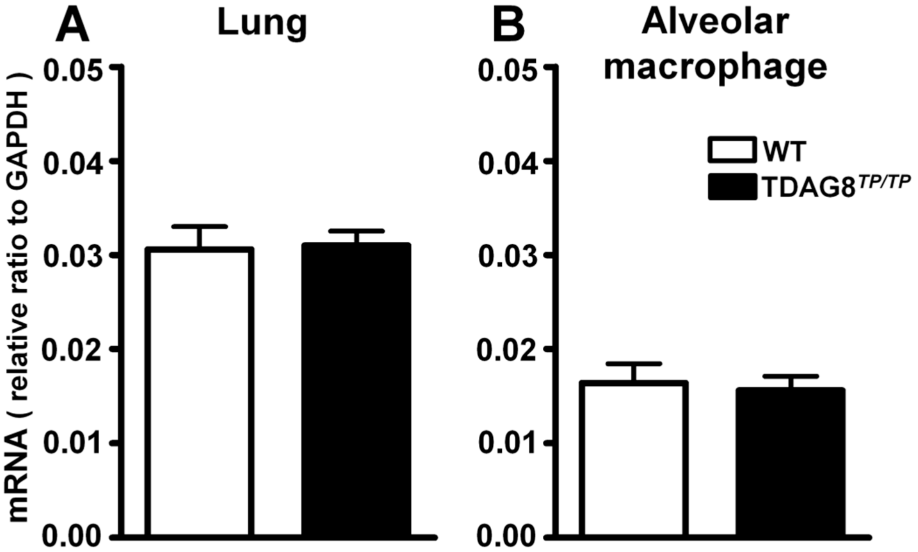

Figure S2. CD14 mRNA expression in lungs and alveolar macrophages. The mRNA expression of CD14 was evaluated by a quantitative real-time TaqMan PCR for lung tissues (**A**) and alveolar macrophages (**B**) in WT and TDAG8-deficient mice.

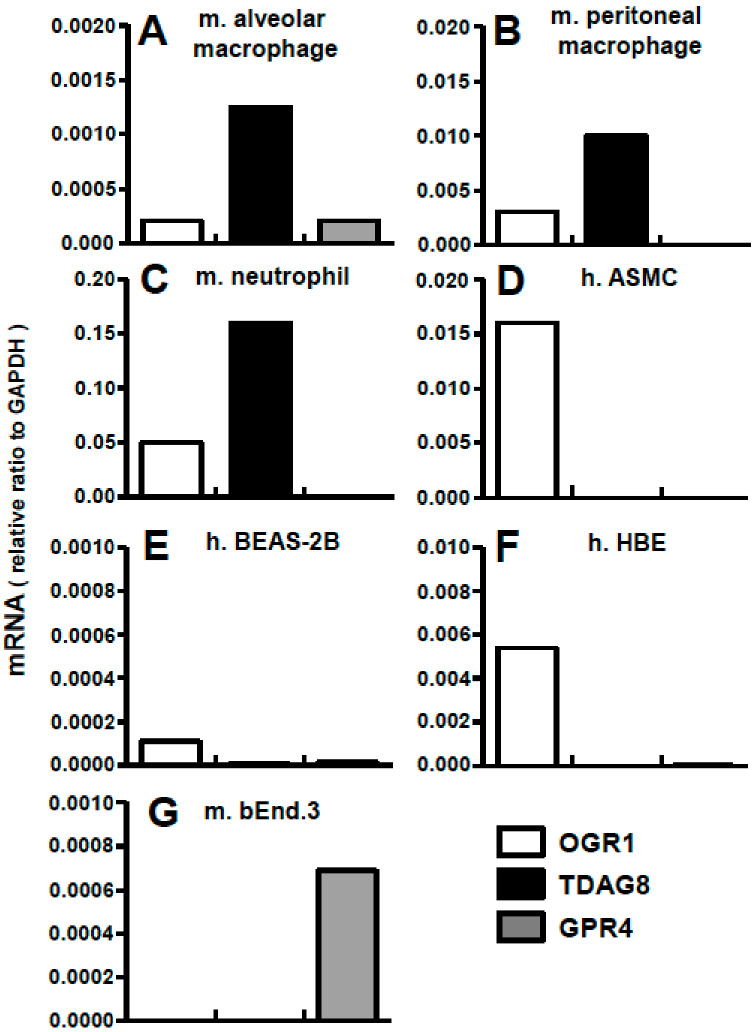

Figure S3. Proton-sensing GPCR mRNA expression in various types of cells. The mRNA expressions of proton-sensing GPCRs were evaluated by a quantitative real-time TaqMan PCR. Cell types are as follows: (**A**) mouse alveolar macrophages (Figure 2C in the present study); (**B**) mouse peritoneal macrophages (data from Mogi *et al.* [10]); (**C**) human neutrophils (data from Murata *et al.* [11]); (**D**) h.ASMC (human airway smooth muscle cells, data from Ichimonji *et al.* [24]); (**E**) h.BEAS-2B (human bronchial epithelial cells, unpublished results); (**F**) h.HBE (human bronchial epithelial cells, unpublished results) and (**G**) m.bEnd.3 (mouse brain endothelial cells, unpublished results). Results are expressed as a ratio relative to GAPDH.
